# Long-term outcomes of cryoablation for biopsy-proven T1 stage renal cell carcinoma

**DOI:** 10.1186/s12957-022-02752-6

**Published:** 2022-09-06

**Authors:** Shangqing Song, Qing Yang, Chengyuan Gu, Guopeng Yu, Bao Hua, Xin Gu, Linhui Wang, Zhong Wang, Guohai Shi, Bin Xu

**Affiliations:** 1 Shanghai Ninth People’s Hospital, Shanghai Jiaotong University School of Medicine, Shanghai, 200011 China; 2 Fudan University Shanghai Cancer Center, Shanghai, 200032 China; 3 Shanghai Changhai Hospital, Naval Medical University, Shanghai, 200433 China

**Keywords:** Renal cell carcinoma, Cryoablation, Laparoendoscopic single-site, Percutaneous

## Abstract

**Background:**

To summarize our clinical experience of cryoablation in renal cell carcinoma (RCC) of Chinese population and to evaluate the long-term outcomes of laparoendoscopic single-site (LESS) cryoablation (LCA) as well as percutaneous CT-guided cryoablation (PCA) for biopsy-proven T1a and T1b RCC.

**Methods:**

This was a multi-center, retrospective study investigating T1 stage RCC patients from 2011 to 2021. The patients were treated by LCA or PCA according to individual situation. Overall survival (OS), cancer-related survival (CSS), and progression-free survival (PFS) were evaluated for oncological outcomes, and kidney function, complications, and hospital stay were used to estimate technical outcomes.

**Results:**

A total of 163 consecutive patients were included. Among them, 59 cases were treated by LCA and PCA was performed in 104 cases. All operations were processed successfully. Mean diameter of the mass was (2.9±1.4) cm; median blood volume was 45ml (10~200 ml). The mean operation time was 84.0 ± 24.5 min. The median postoperative hospital stay was 3 days (1~6 days). Compared with LCA, procedure time of PCA was shortened, the volume of bleeding was reduced, and the hospital stay was decreased. The overall adverse events rate was 9.8% (16/163). The mean preoperative and postoperative eGFR of LCA were 77.6±15.3 ml/min and 75.6±17.4 ml/min, respectively. Analogously, the values of PCA were 78.7±12.9 ml/min and 76.7±14.3 ml/min. Mean follow-up time was 64.2 ± 30.2 months (range, 7–127 months). Local recurrence was observed in 13 patients (8.0%), 4 (6.8%) cases of LCA and 9 (8.7%) cases of PCA. PFS at 5 and 10 years were 95.5% and 69.2% for LCA and 96.7% and 62.8% for PCA. In total, 26 patients (16.0%) (11 patients from LCA and 15 from PCA) died throughout the follow-up period. OS at 5 and 10 years were 93.8% and 31.4% for LCA, and 97.4% and 52.7% for PCA. Six patients (3.7%) (3 cases from LCA and 3 from PCA) died of metastatic RCC. CCS for LCA were 98.0% and 82.8% at 5 and 10 years, while the data were 100% and 86.4% for PCA.

**Conclusion:**

LCA and PCA for T1 stage RCC provides satisfactory long-term oncological and renal function preservation outcomes, with acceptable complication rates.

## Background

The diagnostic rate of stage T1 RCC has been improved significantly over the past two decades owing to the widespread application of imaging technologies, including CT and MRI [1]. About 70% new kidney tumors are diagnosed as T1 stage [2]. Extirpative surgery (radical and partial nephrectomy) remains the first-line management for early stage RCC. Ablative techniques including radiofrequency ablation (RFA) and cryoablation (CA) have emerged as alternatives for patients with RCC which can achieve the three primary objectives: oncologic efficacy, nephron preservation, and low morbidity [3]. For patients with severe comorbidities that may preclude extirpative surgery, ablative therapy is regarded as the most appropriate treatment [4].

Cryoablation can be performed via either a laparoscopic (LCA) or a percutaneous approach (PCA). They represent minimally invasive alternatives to minimally invasive partial nephrectomy by laparoscopy or robot-assisted laparoscopy [5, 6]. It has been proved that LCA and PCA have respective advantages and disadvantages. As PCA for RCC is often performed percutaneously under CT guidance without general anesthesia, it costs less and recovers fast as long as ablation is performed successfully [7, 8]. However, safety and oncologic efficacy of PCA may be potential problems since it is not performed under direct visualization. In contrast, the major superiority of LCA is accurate placement of the probes under direct visualization [8], while it costs more compared with PCA.

Despite the mounting number of studies supporting the use of ablation for T1 RCC, most of them have limitations including small number of patients, lack of histologic proof, and/or short follow-up time.

The aim of the present multi-center study is to sum up 10-year clinical experience of LCA and PCA in RCC of Chinese population and to construe the therapeutic indication and oncologic outcomes of cryoablation for T1 RCC.

## Methods

### Patients

The clinical and pathological data of 163 patients who were treated by PCA or LCA between 2011 and 2021 in Shanghai Ninth People’s hospital (*N* = 55), Shanghai Changhai hospital (*N* = 46), and Fudan University Shanghai Cancer Center (*N* = 62) were analyzed retrospectively. Patients’ demographics including age, gender, tumor location, tumor size, R.E.N.A.L. score, procedure time, follow-up period, the presence of complications, hospital stay, renal function, and survival were analyzed. This study was approved by the Ethics Committee of the Scientific and Ethical Committee of Shanghai Jiaotong University and Fudan University. In addition, informed consent was obtained from all participants.

### Cryoablation protocols

#### Surgery equipment

TriPortTM LESS surgery system and 30° 5mm integrated digital laparoscopic system were used for LCA. The system adopts the 4-probe cryo instrument from EndoCare, the US. The cryo probe is directly percutaneously punctured under laparoscopic or CT guidance. The pressure of argon is 60 PSI and the pressure of the helium is 250 PSI.

#### Method

According to the characteristics of the tumor location, the surgical risk and the patient's willingness, PCA or LCA were performed. The cryoablation procedures including PCA and LCA were operated as described previously [1]. For a patient with familial clustered renal cancer with multiple tumors in both kidneys (left kidney with 4 tumors, and 2 tumors on the right side), tumors were treated on both sides at the same time by LCA for the right kidney and radical nephrectomy for the left kidney. For patients who recurred during follow-up period after cryoablation, laparoscopic nephrectomy or re-cryoablation was performed depending on patients’ willingness or surgeons’ experience.

### Follow-up and outcome evaluation

The follow-up protocols for evaluating the biopsy results and assessing the general recovery status were similar between LCA and PCA groups. All patients underwent CT or MRI examination at the first month post-operation, and then every 3 months during the first post-operation year, and thereafter annually. Complete ablation was defined as continuous size reduction of the target RCC on all subsequent non-enhanced CT or MR images [9, 10]. The calculated eGFR was used to evaluate the renal functional outcomes. To assess the survival rates, the CSS, OS, and PFS rates were analyzed for all patients.

### Statistical analysis

The OS, CSS, and PFS rates were estimated using Kaplan-Meier’s analysis. Each categorical value was evaluated using a paired or an unpaired Student’s *t*-test. All statistical analyses were performed using SPSS software, version 26.0 (IBM Corp, NY, USA), and Graphpad Prism7 (San Diego, CA, USA). The threshold of statistical significance was *p*<0.05.

## Results

### Patient and tumor characteristics

Among 163 patients with T1 stage RCC, 59 cases were treated by LCA and PCA was performed in 104 cases. The majority of lesions treated were T1a (142/163, 87%) and the rest were T1b (21/163, 13%). There were 3 patients suffered with bilateral renal tumors, 1 case was operated with unilateral PCA and contralateral partial nephrectomy; 1 case underwent LCA of multiple renal tumors on one side, and contralateral radical nephrectomy; the other case was treated by PCA for bilateral renal tumors. All 163 cases were pathologically confirmed as RCC, of which 119 cases (73%) were clear cell carcinoma, 20 cases (12%) were chromophobe cell carcinoma, and 24 cases (15%) were papillary cell carcinoma. The characteristics of all patients and tumors are shown in Table 1.

**Table 1:** Patient and tumor characteristics

Patient characteristics	*N*(%)	Tumor characteristics	*N*(%)
Age (years)		Maximum tumor diameter (cm)	
Mean ± SD	69 ± 15	Mean ± SD	2.9 ± 1.4
Range	37–84	Range	1.5–4.9
Sex		Tumor histology	
Male	91(56)	Clear cell RCC	119(73)
Female	72(44)	Papillary RCC	24(15)
Previous nephrectomy		Chromophobe RCC	20(12)
No	154(94)	Laterality	
Yes	9(6)	Right	84(51)
ECOG performance status		Left	76(47)
0	118(72)	Bilateral	3(2)
1	45(28)	Tumor location	
Comorbidities		Exophytic	89(54)
Hypertension	95(58)	Parenchymal	52(32)
Diabetes	48(29)	Mixed	17(11)
Cardiac disease	37(23)	Central	5(3)
Chronic kidney disease	33(20)	R.E.N.A.L. score	
Cryoablation approach		4–6	67(41)
LCA	59(36)	7–9	88(54)
PCA	104(64)	10–12	8(5)
		T stage	
		T1a	142(87)
		T1b	21(13)

*SD* standard deviation, *RCC* renal cell carcinoma, *ECOG* Eastern Cooperative Oncology Group

### Operation parameters

**Fig. 1:**
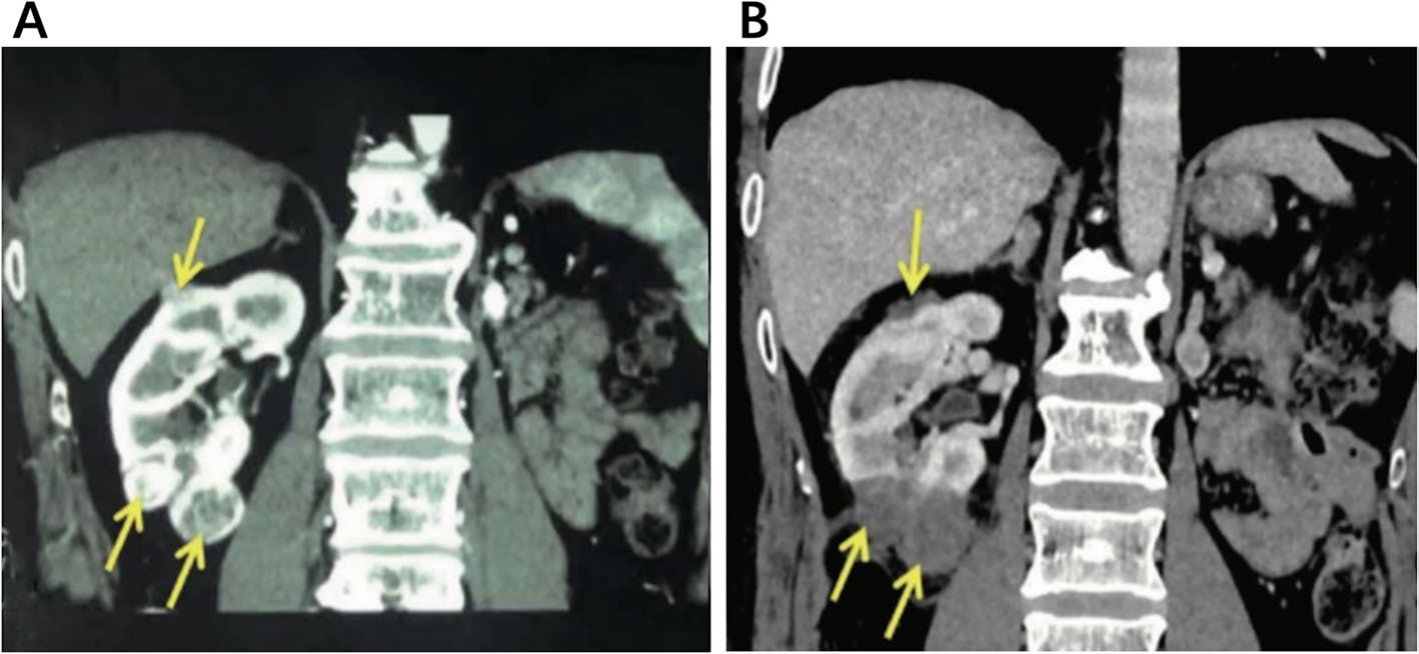
Before and after cryoablation in patients with renal cancer. **A** Preoperative contrast enhanced CT showed nonuniform enhancement of 3 tumors in the upper and lower poles of the right kidney (indicated by arrows). **B** Contrast CT showed no enhancement 1 week after cryoablation (shown by the arrows), indicating complete ablation of tumors

All operations were successfully performed, and no additional incisions were added for LCA. Re-examination of kidney CT scans at the first month after surgery showed that all lesions were low-density without enhancement. Compared with the preoperative CT imaging, it indicated that the tumor had completely subsided (Fig. 1). The median blood loss volume was 45ml (10~200ml). The mean operation time was (84.0±24.5) min. The median postoperative hospital stay was 3 days (1~6 days). Compared with LCA, procedure time of PCA was shortened, the volume of bleeding was reduced, and the hospital stay was decreased (all *p*<0.01) (Table 2).

**Table 2:** Comparison of PCA and LCA

	LCA (*n* = 59)	PCA (*n* = 104)	*p*-value
Procedure time (min)	106.0 ± 21.2	68.1 ± 9.2	<0.01
Blood loss (ml)	60(30~200)	20(10~40)	<0.01
Hospital stay (days)	3(2.5~6)	1(1~3)	<0.01
Adverse events (%)	5(8.5)	11(10.6)	
Urinary tract infection (%)	3(5.1)	3(2.9)	
Large retroperitoneal hematoma (%)	0	3(2.9)	
Small perinephric hematoma (%)	2(3.4)	5(4.8)	

### Adverse events and renal function

**Fig. 2:**
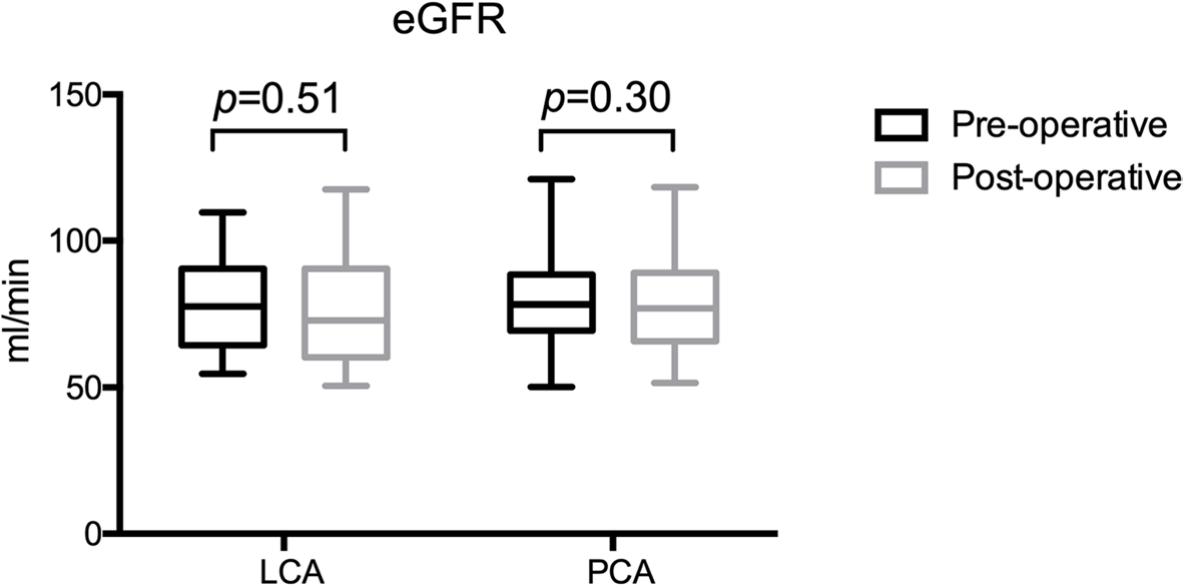
Box-plot representation of pre- and post-procedural values of eGFR

The overall adverse event rate was 9.8% (16/163) (5 cases of LCA and 11 of PCA) (Table 2). Of those, 5.5% (9/163) were classified as Clavien–Dindo grade II and included 6 urinary tract infection, 3.7% successfully treated with antibiotics and 3 cases (PCA) of large retroperitoneal hematoma with active extravasation at CT angiography (1.8%), which resolved without further sequelae. The remaining 7 adverse events were classified as Clavien–Dindo grade I with self-resolved small perinephric hematoma (4.3%) (5 cases of PCA and 2 of LCA). None of the patients had intraoperative or postoperative complications classified as Clavien–Dindo grade III or greater. The mean preoperative and postoperative eGFR of LCA were 77.6±15.3 ml/min and 75.6±17.4 ml/min, respectively. Analogously, the eGFR values of PCA were 78.7±12.9 ml/min and 76.7±14.3 ml/min, respectively (Fig. 2). No statistically significance was revealed between pre- and postoperation, and none of the patients require dialysis during the follow-up period.

### Survival

**Fig. 3:**
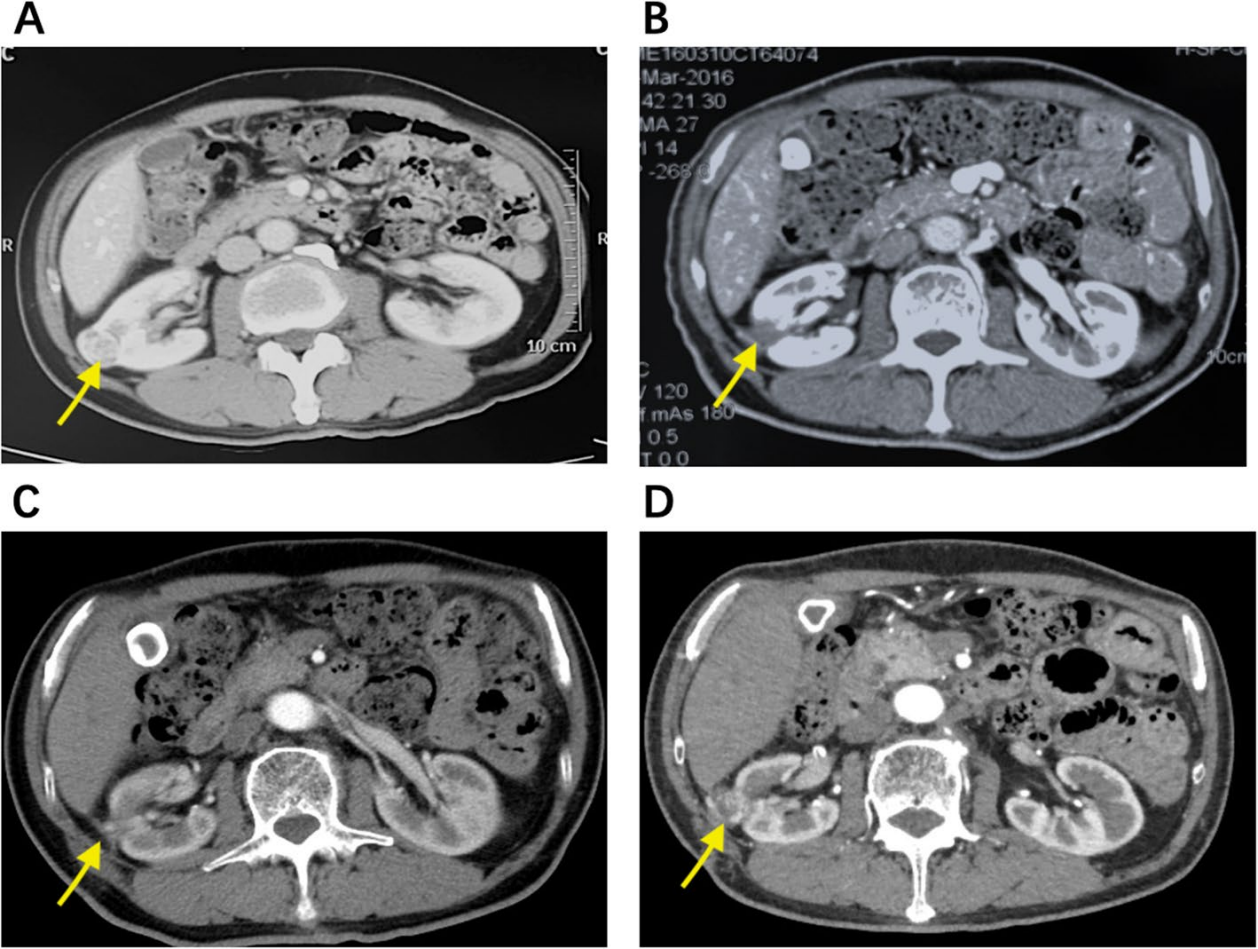
Ablation and recurrence after PCA (shown by arrows). **A** Contrast-enhanced CT before cryoablation, showing nonuniform enhancement of tumor. **B** One week after ablation, the tumor showed no enhancement, indicating that the tumor has been completely ablated. **C** Twenty-four months after the operation, the tumor still has no enhancement, indicating no recurrence. **D** Sixty-one months after the ablation, a tumor appeared at the original location with enhancement, indicating recurrence

**Fig. 4:**
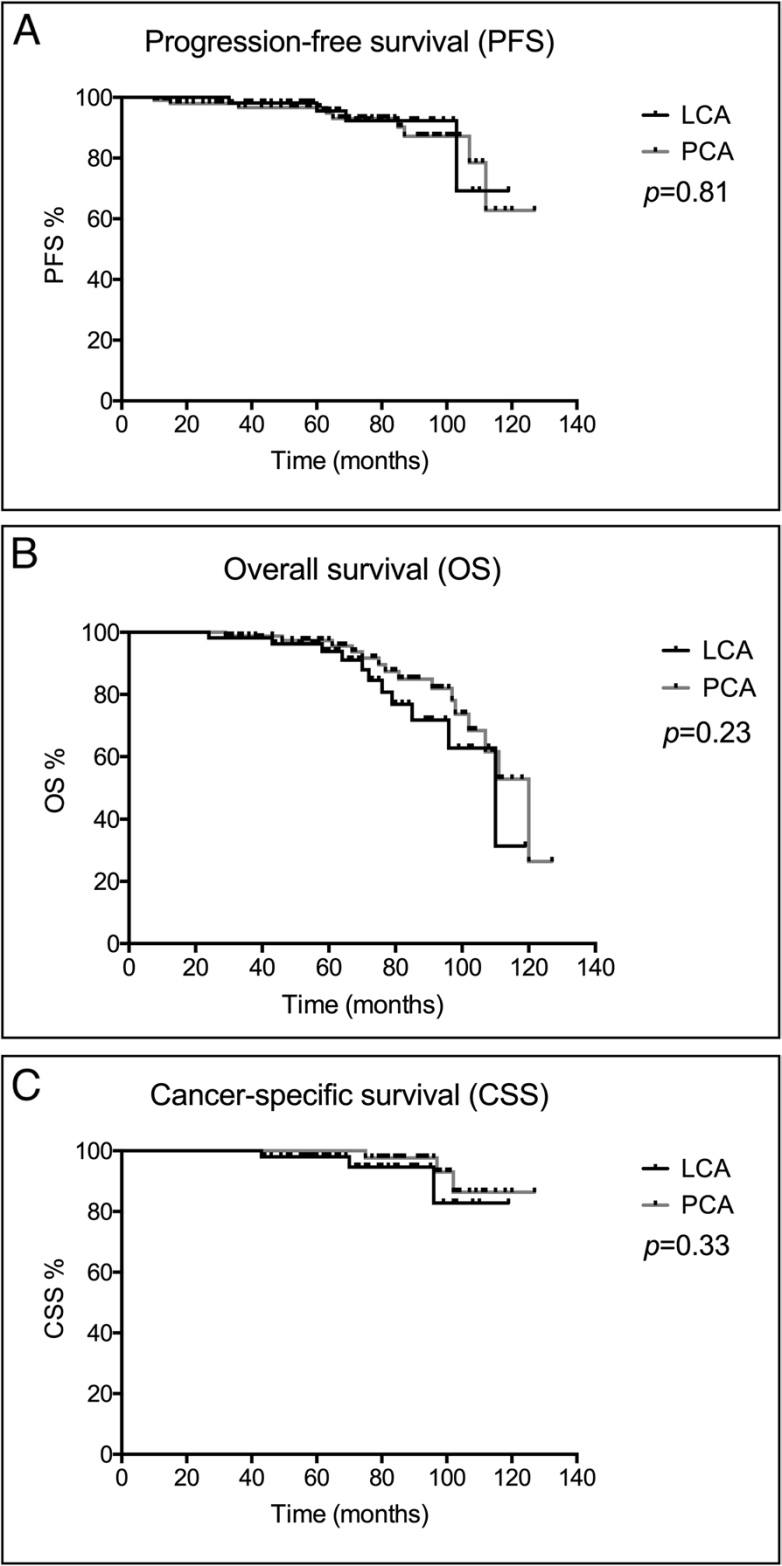
Kaplan–Meier plots of patients’ survival. **A** Progression-free survival. **B** Overall survival. **C** Cancer-specific survival

Mean follow-up duration was 64.2 ± 30.2 months (7–127 months). Local recurrence was observed in 13 patients (8.0%), 4 (6.8%) cases of LCA and 9 (8.7%) cases of PCA. Among these patients, 10 of them were retreated by PCA. In 2 patients, laparoscopic nephron-sparing nephrectomy (LNSS) was performed due to recurrence (Fig. 3), while in one patient with recurrence, laparoscopic radical nephrectomy (LRN) was performed. PFS at 5 and 10 years were 95.5% (56) and 69.2% (41) for LCA and 96.7% (100) and 62.8% (65) for PCA (Fig. 4A). In total, 26 patients (16.0%) (11 patients from LCA and 15 from PCA) died throughout the follow-up period. According to Kaplan–Meier analysis, 5 and 10 years’ estimated OS was 93.8% (55) and 31.4% [11] for LCA, and 97.4% (101) and 52.7% (55) for PCA (Fig. 4B). Six patients (3.7%) (3 cases of LCA and 3 cases of PCA) died of metastatic RCC. CCS for LCA were 98.0% (58) and 82.8% (49) at 5 and 10 years’ follow-up, while it was 100% (104) and 86.4% (90) at 5 and 10 years for PCA (Fig. 4C). In the remaining 20 patients who did not die of RCC, deaths were attributed to cardiovascular events (10 cases), respiratory failure (4 cases) accidents (3 cases), colon cancer (2 cases), and liver cancer (1 case).

## Discussion

Cryoablation was first recognized as a technique for the treatment of prostate cancer in urology; later, it was used to treat solitary lesions of the kidney in patients with renal insufficiency or high-risk patients who could not tolerate an extirpative procedure; afterwards, laparotomy cryoablation was used to treat renal tumors [12]. Subsequently, LCA was developed for the treatment of early stage RCC owing to its advantages such as delivery of the energy probe in a controlled fashion under direct vision [13] . To further reduce trauma to the patient, PCA is performed under sedation, thus avoiding entry into the peritoneal [14]. Although either LCA or PCA has its own advantages, PCA is more frequently chosen due to minimal invasiveness and quick postoperative recovery as compared with LCA. PCA was more frequently used for treating kidney tumor located in the posterior and/or lateral aspect of the kidney due to the limited anatomical structure. However, with successful utilization of adjunctive displacement maneuvers, PCA may be selected as a routine treatment for anterior kidney tumors [15].

The R.E.N.A.L. nephrometry scoring system has been used to evaluate the tumor complexity and to choose an appropriate treatment for renal masses [16] . KIM et al. [17] and Zargar et al. [18] suggest that R.E.N.A.L. scores of PCA are higher than those of LCA, implying that tumors treated with PCA are more complex than those treated with LCA. Nevertheless, Finley et al. [11] reported that there was no significant difference in the R.E.N.A.L. score between the two groups. Comparing the R.E.N.A.L. score with complications of cryotherapy showed that the R.E.N.A.L. score is associated with complications and tumor recurrences after cryotherapy [17, 19]. In addition, perioperative complications and hospital stay are also used to assess the merits and demerits between PCA and LCA. One study by David et al. [11] showed that PCA was associated with fewer complications than LCA. However, in the present study, we found that PCA was more likely to contribute perinephric hematomas, although generally no intervention is required. Consistent with our result, procedure time and hospital stay in patients undergoing PCA are significantly shorter than that in patients treated by LCA, which due to the avoidance of general anesthesia and lesser skin incisions [5]. All these results demonstrate that postoperative recovery in PCA patients is faster than that in LCA patients under the same conditions. Nevertheless, in theory, there are limitations of PCA, including injury of the surrounding tissues and organs such as intestine, and it is difficult to place the cryo-probe in the ideal position due to substantial breath or uncooperative patients, although these events did not happen in our study. As a result, we recommend if large blood vessel, renal pelvis, ureter, or other abdominal organs are nearby the tumor, LCA should be preferential. In the present study, tumor location was considered as one of the impact factors for adopting PCA or LCA approach. For PCA, tumors were located at exophytic (68 cases, 65%) part and parenchymal (36 cases, 35%) part, while tumors from LCA group were located at exophytic (21 cases, 36%) part, parenchymal (16 cases, 27%) part, central part (5 cases, 8%), and mixed sites (17 cases, 29%). During the follow-up period of all cases, no significant renal dysfunction was found, suggesting that cryoablation has little effect on the remaining normal kidney tissue.

In the current study, the OS, CSS, and PFS rates were comparable to that of laparoscopic or open partial nephrectomy in patients with T1 RCC [20]. PCA and LCA showed no significant difference in survival time. Nevertheless, the 2018 National Comprehensive Cancer Network guidelines for kidney cancer stated that ablative techniques have been associated with increased risk of local recurrence [21]. More recently, the European Association of Urology guidelines for RCC recommended that “When radiofrequency ablation, cryoablation, and active surveillance are offered, inform patients about the higher risk of local recurrence and/or tumor progression”. Meanwhile, a recent study reported that PCA yielded a 10-year disease-specific survival of 94%, equivalent to that reported after radical or partial nephrectomy [22]. Moreover, MRI-guided PCA of RCC is associated with acceptable complication rates and high estimates of survival at 5 years, which are substantially similar to those derived from series using CT guidance [23]. Regarding the technic of cryoablation, high local efficacy could be obtained when tumor lesions are ablated with adequate ablative margin in a carefully controlled way [24]. Associating our results with recent studies, cryoablation should not be considered as shorter CSS, PFS, or OS survival of T1 stage RCC patients. Factors associated with oncological outcomes always dealt with characteristics of patients and tumors.

Recently, one meta-analysis revealed that renal function preservation and complication rates were not different between partial nephrectomy and cryoablation therapy in patients with both cT1a and cT1b renal tumors. When partial nephrectomy was compared to PCA, the recurrence rate was not statistically different in cT1a patients, and cryoablation would be an alternative treatment for select patients with cT1 renal tumor [25]. In another meta-analysis, Yoon et al. [26] reported that the incidence of both overall and severe complications was not different between the 2 treatment techniques comparing robot-assisted partial nephrectomy to ablation therapy (including LCA and PCA, PRFA (percutaneous radiofrequency ablation)) in patients with cT1 renal tumors. Moreover, candidates for active surveillance are often the same patients for whom ablation therapy is considered.

Several studies compared patients with cT1a tumors treated by LCA or PCA and RFA. In both CA and RFA, complication and recurrence rates were similar between patients treated laparoscopically and percutaneously [25]. However, one meta-analysis evaluated 47 studies to compare RFA to cryoablation and determined that tumor progression was significantly higher after radiofrequency ablation than after cryoablation [27].

It follows that cryoablation therapy has an important role in the treatment of small renal tumors and shows comparable oncological and functional outcomes to partial nephrectomy. In a subset of advanced age, comorbid, and high-risk surgical RCC patients, especially those who are reluctant or have failed to perform active surveillance, cryoablation therapy might be superior to partial nephrectomy.

Despite the advantages of cryoablation for T1 stage RCC showed from our study, there are several limitations. First, it was a retrospective study and the purpose was not the comparison to other treatment strategies, including active surveillance and nephron-sparing surgeries. Second, the number of patients were not enough yet for an unassailable consequence. Finally, the small number of T1b cases herein treated precludes any significative comparison of outcomes between the subgroups of T1a and T1b.

## Conclusion

Both PCA and LCA for T1 RCC showed a rather high long-term tumor control with a low frequency of complications. The cryoablation has a good clinical application prospect especially for the patients with severe or multiple comorbidities and is accompanied by renal insufficiency, with bilateral renal cancer or solitary renal cancer.

## Data Availability

The data that support the findings of this study are available on request from the corresponding author.

## Abbreviations

RCC: Renal cell carcinoma
LESS: Laparoendoscopic single-site
LCA: Laparoendoscopic single-site cryoablation
PCA: Percutaneous CT-guided cryoablation
OS: Overall survival
CSS: Cancer-related survival
PFS: Progression-free survival
RFA: Radiofrequency ablation
PRFA: Percutaneous radiofrequency ablation
